# Analytical and experimental position stability of the abutment in different dental implant systems with a conical implant–abutment connection

**DOI:** 10.1007/s00784-012-0786-1

**Published:** 2012-07-22

**Authors:** Wiebke Semper-Hogg, Silvan Kraft, Sebastian Stiller, Juergen Mehrhof, Katja Nelson

**Affiliations:** 1 Department of Oral and Maxillofacial Surgery, University Hospital Freiburg, Hugstetter Str. 55, 79106 Freiburg, Germany; 2 Department of Radiology, Technical University Munich, Ismaninger Str. 22, 81675 München, Germany; 3 Zahntechnik Mehrhof GmbH, Reuchlin Str. 10-11, 10553 Berlin, Germany

**Keywords:** Rotational freedom, Vertical displacement, Conical, Positional index, Hexagon, Cam–groove design

## Abstract

**Objectives:**

Position stability of the abutment should be investigated in four implant systems with a conical implant–abutment connection.

**Materials and methods:**

Previously developed formulas and an established experimental setup were used to determine the position stability of the abutment in the four implant systems with a conical implant–abutment connection and different positional index designs: The theoretical rotational freedom was calculated by using the dimensions of one randomly selected implant per system for approximated geometric models. Experimentally, the rotation, the vertical displacement, and canting moments of the abutment after multiple repositioning and hand tightening of the abutment screw were investigated.

**Results:**

The experimental rotation and vertical displacement differed between the implant systems tested. The analytical and experimental results for the rotation of the abutment clearly deviated in the three implant systems.

**Conclusions:**

Malpositioning of the abutment was possible in all the implant systems tested. Deviating theoretical and experimental results suggest high manufacturing tolerances during fabrication of the implant components.

**Clinical relevance:**

Position stability of the abutment is essential for precisely fitting implant-supported superstructures.

## Introduction

Mechanical complications still exist in implant dentistry including fractures or mobility of the superstructure and the abutment screw [1, 2]. These complications have a multifactorial etiology [3–5]. Current studies indicate that position stability of the implant–abutment connection (IAC) is a decisive factor for prosthesis misfit [6, 7] and mechanical complications [8]. Position stability of the implant–abutment connection is essential, since multiple repositioning of the implant components is necessary during fabrication of the superstructure by the dental technician and the dentist [9, 10].

Rotational position stability of IACs with different positional index designs has been investigated [11–16]. The results indicate that the rotational freedom of different positional indices of the second and third generation is similar to that of hexagonal indices of the first generation [16]. Theoretical calculations show that the position stability depends on the geometric design of the index and the manufacturing tolerances [14, 15].

Conical connections were developed to achieve a friction-based fit of the implant components [16–20]. Little has been published about the repositioning accuracy of the abutment in conical implant–abutment connections [16, 21].

In the present study, position stability of the abutment in various implant systems with a conical implant–abutment connection was examined in vitro, and its rotational freedom was theoretically calculated. Working hypotheses were that the theoretical optimal result for the rotational freedom of the index corresponds to the findings of the experiment and that the experimental results do not differ between the implant systems tested.

## Materials and methods

**Fig. 1:**
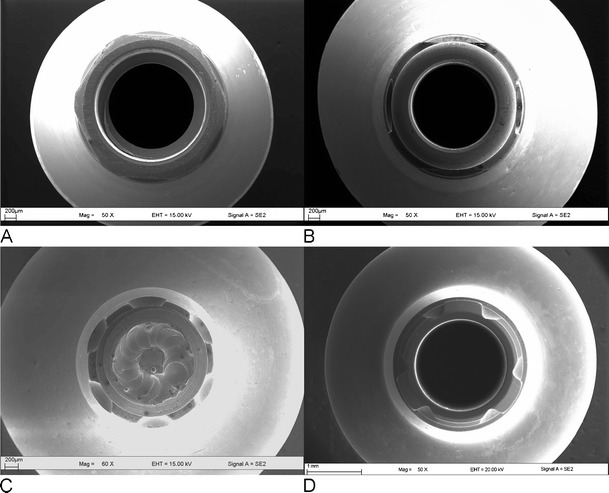
Abutments showing the positional index designs. **a** S1: Nobel Active. Conical implant–abutment connection with a cone angle of 12° and an internal hexagonal positional index. **b** S2: Bone Level. Conical implant–abutment connection with a cone angle of 15° and cams and grooves. **c** S3: Ankylos C/X. Conical implant–abutment connection with a cone angle of 5.7° and six cams and grooves. **d** S4: Conelog. Conical implant–abutment connection with a cone angle of 7.5°and three cams and grooves

The position stability of different implant systems with a conical implant–abutment connection was investigated: Nobel Active (S1), Bone Level (S2), Ankylos C/X (S3), and Conelog (S4) (Table 1, Fig. 1). Seven implants and rotation-safe abutments of each implant system were purchased regularly. To allow comparability of the results, implants with similar diameters were ordered (Table 1).

**Table 1:** Specifications of the implants and abutments used

System	Ident.	Article no.	Lot no.	Ident.	Mat	Article no.	Lot no.	Manufacturer
Nobel Active (S1)	Internal RP, 4.3 × 11.5 mm	34132	707722	Esthetic abutment	Ti	34199	708540	Nobel Biocare AB, SE-40226 Göteborg, Sweden
			696896				708540	
			696896				708540	
			707023				708540	
			707023				708540	
			707023				708540	
			707023				708540	
Bone Level (S2)	Bone Level, 4.1 RC SLActive, 12 mm	021.4112	K6756	Universal abutment	Ti	022.2104	X3223	Institut Straumann AG, CH-4002 Basel, Switzerland
			F7007				R8701	
			F7007				R8701	
			F7007				R8701	
			F7007				R8701	
			F7007				R8701	
			F7007				R8701	
Ankylos (S3)	C/X Implant, B14∅4.5/L14	170556/31010440	0020039200	Regular/X abutment GH3.0/A0	Ti	31024130	20035835	FRIADENT GmbH, 68229 Mannheim, Germany
			0020034919				20035835	
			0020034919				20035835	
			0020039201				20035835	
			0020039201				20035835	
			0020038909				20035835	
			0020038909				20035835	
Conelog (S4)	Screw-Line ConeLog Implant Promote plus ∅4.3 L11	E-C1062.4311	0000029157	Universal abutment	Ti	C2211.4300	0000028679	Camlog Biotechnologies AG, CH-4053 Basel, Switzerland
			0010024599				0010027056	
			0010024599				0010027056	
			0010024599				0010027056	
			0010024599				0010027056	
			0010024599				0010027056	
			0010024599				0010027056

### Theoretical calculation

#### Calculation of the rotational freedom of the abutment

The measured dimensions of the positional index of one randomly selected implant and abutment per system (Table 1) [15] were used to calculate the maximum rotational freedom of the positional index designs by utilizing previously developed closed-form formulas for approximated geometric models [14, 15]. The clearance between implant and abutment was set at 20 μm according to the previous investigation [15].

### Experimental investigation

#### Experimental setup

An established experimental setup [16] was used. The six implants of each system were fixated in prefabricated stainless steel models that were manufactured to imitate a clinical situation. The implants were fixated in angulations of 0° (implants 1 and 6), 5° (implants 2 and 5), and 15° (implants 3 and 4).

#### Experiment

The experiment was performed as described with the exception that two test persons having different implantological skills dis- and reassembled the implant–abutment test body complexes 20 times each by hand tightening the screw [16]. The test persons were specified as Pe1 (=person with implantological skills) and Pe2 (=person without implantological skills).

#### Statistical analysis

The mean and standard deviation of the experimental values were calculated. Since the mean and median were skewed, the median and quartiles were used for the analysis. The maximum bidirectional displacement was expressed by the maximum range of deviation (most positive value + most negative value).

A two-factorial nonparametric analysis for repeated measurements [22] was used to analyze the influence of the implant system and the test person on the outcomes. The implant system was set as a whole-plot factor and the test person as a split-plot factor.

Statistical analysis was performed using SPSS 17.0 (SPSS Inc., Chicago, IL, USA) and SAS 9.1 (SAS Institute Inc., Cary, NC, USA). A *P* value <0.05 was considered statistically significant.

## Results

### Theoretical calculation

#### Rotation of the abutment

##### Hexagonal positional index design

Using the formula for polygonal positional index designs, a rotational freedom of 3.1° results for S1 (Table 2).

**Table 2:** Values inserted into the previously developed formulas to calculate the rotational freedom of the positional index for the implant systems used

Polygon	*R* (mm), width across vertices	*n*, number of vertices	*C* (mm), clearance between implant and abutment	*α* (°), rotational freedom
S1	1.52	6	0.02	3.1
Cam–groove	*R* (mm), distance of contact point to rotational axis	*δ* (°), angle between R and implant wall	*C* (mm), clearance between implant and abutment	*α* (°), rotational freedom
S2	1.35	2.0	0.02	1.7
S3	1.00	29.5	0.02	2.6
S4	1.15	18.1	0.02	2.1

##### Cam–groove design

The maximum rotational freedom of the systems having a cam–groove connection design was calculated using a universal equation. A theoretical rotational freedom of 1.7° results for S2, of 2.6° for S3, and of 2.1° for S4 (Table 2).

### Experimental investigation

#### Rotation of the abutment

The median rotation of S1 (hexagon) was 1.19° (0.39°, 2.11 °), and the maximum range of rotational movement of the abutment was 7.27°. S2 (cam–groove) showed a similar median rotational displacement of 1.09° (0.79°, 1.39°) with a smaller maximum range of 4.29°. The system S3 (cam–groove) displayed a median rotation of 0.82° (0.33°, 1.95°) showing a maximum range of 6.22°. S4 (cam–groove) showed a median rotation value of 0.25° (0.15°, 0.34°) and a maximum range of 2.14°. The minimal and maximal values for each implant system and test person are shown in Table 3. Rotational displacements of the abutments tested differed significantly between the implant systems (*P* < 0.001) with S4 showing significantly lower rotational freedom than S1, S2, and S3 (all *P* < 0.001). S1, S2, and S3 were not different (S1 vs. S2, *P* = 0.718; S1 vs. S3, *P* = 0.273; S2 vs. S3, *P* = 0.423). The test person did influence the outcome (*P* < 0.001).

**Table 3:** Minimal (min) and maximal (max) experimental rotational and vertical displacement measurement values for each implant system and test person

System/implant	Person 1		Person 2	
Rotation				
	Min (°)	Max (°)	Min (°)	Max (°)
S1	0.07	3.95	<0.01	3.23
1	0.14	3.41	0.02	2.59
2	0.07	3.37	<0.01	1.31
3	0.86	3.88	0.01	1.03
4	0.13	2.29	0.01	3.23
5	1.15	3.95	0.04	0.69
6	0.60	3.28	0.04	0.63
S2	0.38	2.58	0.04	2.50
1	0.55	1.30	0.22	1.40
2	0.38	1.22	0.25	1.24
3	0.44	2.58	0.25	2.50
4	1.00	1.89	0.14	1.89
5	1.25	1.48	0.41	1.62
6	0.61	2.00	0.04	1.39
S3	0.13	3.84	0.02	4.20
1	0.87	3.56	0.02	1.05
2	1.24	3.42	0.04	4.10
3	0.51	3.84	0.29	4.20
4	1.53	3.23	0.05	0.76
5	0.14	1.73	0.14	1.55
6	0.13	3.15	0.20	1.49
S4	<0.01	1.30	<0.01	1.72
1	0.07	0.88	<0.01	0.88
2	<0.01	1.28	0.05	0.93
3	<0.01	1.21	<0.01	0.77
4	0.01	1.17	<0.01	0.39
5	0.02	0.89	<0.01	0.28
6	0.04	1.30	<0.01	1.72

Vertical displacement				
	Min (μm)	Max (μm)	Min (μm)	Max (μm)
S1	<1	21	<1	14
1	<1	13	<1	12
2	<1	12	<1	14
3	<1	19	<1	8
4	<1	21	<1	10
5	<1	11	<1	7
6	<1	7	<1	9
S2	<1	8	<1	8
1	<1	6	<1	5
2	<1	5	<1	5
3	<1	8	<1	7
4	<1	8	<1	8
5	<1	6	<1	6
6	<1	6	<1	6
S3	<1	11	<1	7
1	<1	7	<1	7
2	<1	9	<1	7
3	<1	5	<1	4
4	<1	4	<1	2
5	<1	11	<1	5
6	<1	6	<1	3
S4	<1	25	<1	17
1	<1	10	<1	9
2	<1	9	<1	7
3	<1	6	<1	14
4	<1	25	<1	17
5	<1	13	<1	10
6	<1	10	<1	11

#### Vertical displacement of the abutment

S1 (cone angle, 12°) showed a median deviation of 3 μm (3 μm, 5 μm) with a maximum range of 39 μm. S2 (cone angle, 15°) and S3 (cone angle, 5.7°) showed a similar median vertical displacement with S2, 2 μm (2, 3 μm) and S3, 2 μm (1, 3 μm). Also, the maximum range of vertical displacement was similar: S2, 16 μm and S3, 18 μm. S4 (cone angle, 7.5°) displayed a median vertical deviation of 4 μm (3, 5 μm) with a maximum range of 41 μm. The vertical deviation of the abutment depended on the implant system (*P* = 0.001) (Table 3). The values for S1 vs. S4 and S2 vs. S3 comparing the extent of the vertical deviation did not differ significantly (S1 vs. S4, 0.476; S2 vs. S3, *P* = 0.886). Significant differences were observed for S1 vs. S2 (*P* = 0.002), S1 vs. S3 (*P* = 0.001), S2 vs. S4 (*P* < 0.001), and S3 vs. S4 (*P* < 0.001). The outcome differed significantly between Pe1 and Pe2 (*P* < 0.001).

#### Canting moments of the abutment

The median changes in position were 0.09° (0.04°, 0.18°) for S1, 0.05° (0.03°, 0.08°) for S2, 0.02° (0.01°, 0.11°) for S3, and 0.05° (0.04°, 0.05°) for S4. The maximum range of canting moments was 1.20° (S1), 0.46° (S2), 0.79° (S3), and 0.38° (S4). Position stability related to the canting of the abutment did not differ between the four systems (*P* = 0.167). Significant differences between the results of the two test persons were observed (*P* < 0.001).

## Discussion

The results of the present study show that repositioning of the abutment in all the four implant systems with an internal conical implant–abutment connection varies.

The analytical maximum rotational freedom based on equal clearance values (manufacturing tolerance) varies (1.7°–2.6°) in the cam–groove group (S2–S4); this is due to the geometric variation of *R* and *δ*. Cam–groove butt joint connections with a greater *R* show less rotational freedom [15]. This can be related to the limited sizing inside the implant when the index is placed more apical in conical connections in contrast to the sizing of the index in butt joint connections. As described previously, polygonal antirotational indices show higher rotational freedom than cam–groove connections due to the unfavorable features of their geometric design.

When analyzing the experimental rotational displacement of the abutment, significant differences between the implant systems were observed: The measured values of the Conelog system were significantly different to all other systems tested whereas Nobel Active, Bone Level, and Ankylos C/X showed similar values. Comparing the results to the outcome of a previous experimental study, the maximum rotational displacement of Nobel Active (hexagon) in the experiment is higher than the results for polygonal indices [16] and hexagonal indices in other studies [12, 13, 23]. Internal hexagonal indices such as Nobel Active show less rotational position stability than external indices again due to their smaller sizing (radius of index from rotational axis) [12, 16]. The investigated cam–groove connection designs show an increased rotational displacement in comparison to the Camlog system of the previous study [16] with Bone Level and Ankylos C/X clearly deviating.

In 1996, Binon declared that a possible rotational movement of the abutment of less than 5° assures screw joint stability of the implant–abutment complex in systems with an external hexagonal connection [11]. Further guidelines were not found. The Conelog system meets this standard; the Bone Level system barely meets this requirement. The two other implant systems (Nobel Active and Ankylos C/X) showed higher rotation values. Whether these findings compromise the stability of the conical screw joint needs to be determined; they definitely influence the fit of the superstructure [6], and a passive fit cannot be achieved.

The analytical approach of the present study aimed to show the rotational freedom of different geometric designs under idealized conditions, while the experimental part shows the possible reproducibility of the abutment position after physical repositioning. The experimental rotational freedom of the implant systems does not reflect the possible rotational freedom from the left to the right horizontal stop; if this was simulated, it would probably lead to higher rotational values. Shape irregularities (e.g., flattening of the vertices in polygons) may also influence the outcome. Manufacturing tolerances during fabrication of the implant components have a major influence on the rotational freedom in all index designs [14, 15]. Relating the theoretical findings to the experimental results shows that the rotational freedom of Nobel Active, Bone Level, and Ankylos C/X is approximately twice as large as calculated by the analytical approach. This suggests high manufacturing tolerances irrespective of the geometric design of the index. For the Conelog system, high-precision manufacturing can be assumed as the theoretical and experimental results were similar.

Canting moments of the abutment were not different between the four implant systems. They were of minimal extent as observed in the previous study [16].

Investigating the vertical displacement of the abutment after multiple repositioning and hand tightening resulted in significant differences between the systems, with Nobel Active and Conelog showing a different behavior from Bone Level and Ankylos C/X. Although the median values were similar, the maximum range of values clearly deviated. Nobel Active and Conelog displayed a higher vertical displacement of the abutment comparable to the systems with a conical connection design (Straumann Tissue Level and Astra Tech) from a former study [15]. For Bone Level and Ankylos C/X, a reduced vertical displacement of the abutment was observed. Still, their extent of vertical displacement is higher than in butt joint connections [16, 24, 25]. A vertical displacement is natural in conical connections and essential for a friction fit. The vertical position of the abutment is not only influenced by the cone angle, as the systems with the most acute (Ankylos C/X) and most obtuse (Bone Level) angle show comparable results. Other factors including the manufacturing tolerances (Semper et al., unpublished observation) and the torque value applied [21] may be of importance for the present results. The present approach is based on hand tightening of the screw, as suggested by the manufacturers until definitive seating of the abutment. Investigations showed that there is a wide variability of torque values when the abutment screw is manually tightened [26, 27]. Achieving predictable and repeatable torque values using hand tightening seems to be impossible. Therefore, the vertical position of the abutment in conical implant–abutment connections can vary between repeated screw tightening. Further studies should be performed using a torque wrench to investigate the final positioning of the abutment.

## Conclusion

Malpositioning of the abutment was possible in all systems with a conical implant–abutment connection tested. The experimental rotational freedom and the vertical displacement of the abutment differed between the implant systems. In three implant systems, the theoretical rotational freedom clearly deviated from the experimental results suggesting high manufacturing tolerances diminishing position stability of the abutment.
